# Differentiation of neurons from neural precursors generated in floating spheres from embryonic stem cells

**DOI:** 10.1186/1471-2202-10-122

**Published:** 2009-09-24

**Authors:** Huawei Li, Hong Liu, C Eduardo Corrales, Jessica R Risner, Jeff Forrester, Jeffrey R Holt, Stefan Heller, Albert SB Edge

**Affiliations:** 1 Department of Otolarygology, EENT Hospital of Shanghai Medical College of Fudan University, Institute of Biomedical Sciences, Fudan University Shanghai, 200031, PR China; 2 Departments of Otolaryngology-HNS and Molecular and Cellular Physiology, Stanford University School of Medicine, Stanford, CA 94305, USA; 3 Department of Otology and Laryngology, Harvard Medical School, Boston MA 02115, USA; 4 Eaton-Peabody Laboratory. Massachusetts Eye and Ear Infirmary, Boston, MA 02114, USA; 5 Program in Speech and Hearing Bioscience and Technology, Division of Health Science and Technology, Harvard and MIT, Cambridge, MA 02139, USA; 6 Department of Neuroscience, University of Virginia, Charlottesville, VA 22908, USA; 7 Department of Otolaryngology, University of Virginia, Charlottesville, VA 22908, USA

## Abstract

**Background:**

Neural differentiation of embryonic stem (ES) cells is usually achieved by induction of ectoderm in embryoid bodies followed by the enrichment of neuronal progenitors using a variety of factors. Obtaining reproducible percentages of neural cells is difficult and the methods are time consuming.

**Results:**

Neural progenitors were produced from murine ES cells by a combination of nonadherent conditions and serum starvation. Conversion to neural progenitors was accompanied by downregulation of *Oct4 *and *NANOG *and increased expression of *nestin*. ES cells containing a GFP gene under the control of the *Sox1 *regulatory regions became fluorescent upon differentiation to neural progenitors, and ES cells with a tau-GFP fusion protein became fluorescent upon further differentiation to neurons. Neurons produced from these cells upregulated mature neuronal markers, or differentiated to glial and oligodendrocyte fates. The neurons gave rise to action potentials that could be recorded after application of fixed currents.

**Conclusion:**

Neural progenitors were produced from murine ES cells by a novel method that induced neuroectoderm cells by a combination of nonadherent conditions and serum starvation, in contrast to the embryoid body method in which neuroectoderm cells must be selected after formation of all three germ layers.

## Background

Embryonic stem cells are potentially a source of all cell types but the problem of converting these cells to desired cell types with high efficiency *in vitro *has remained a challenge in transplantation and developmental biology [1,2].

Since embryoid bodies contain a mixture of ectodermal, mesodermal and endodermal germ layers, differentiation of stem cells to neurons has usually been achieved by protocols that enrich ectoderm derivatives, as a first step in the production of neural progenitors. These attempts to influence cell fate decisions to obtain cells of the ectodermal layer involve the use of neuralizing signals that can be derived from feeder cells, addition of growth factors, or growth supplements added to the medium [3-8]. ES cell-derived progenitors with characteristics of ectoderm have been converted to neurons by several methods, and in many instances further steps have been designed to specify individual neuronal types [1,8-10]. Neural progenitors from ES cells also give rise to glial cells [11,12] and sensory cells [13]. However, the protocols are often complicated and can be expensive and time consuming, requiring several weeks of specific culture conditions to obtain cells that have the capacity to differentiate into neurons.

We have found that murine ES cells differentiate into neural progenitors in defined medium in spheres, and we have been able to obtain neurons fairly rapidly with little manipulation and with few expensive reagents. This procedure takes advantage of the differentiation to neural lineage cells in floating spheres as well as the survival of these neural precursors in serum-free medium. We have used ES cell lines that have been engineered to express reporters for the progenitor state and the fully differentiated state to show that these cells give rise to the desired differentiated cell types. The protocol does not use feeder cells or embryoid body formation and is similar to the monolayer expansion and differentiation protocol of Ying et al. [14], but differs from that protocol by the use of floating spheres which substantially increases the yield of neural progenitors. The progenitors obtained from the serum starvation and suspension culture give rise to neurons, glial cells, and oligodendrocytes. They can be expanded by adherent culture with bFGF and retain their capacity to differentiate into neurons. This protocol will be useful for rapid generation of large numbers of neural progenitors that can be used in studies of transplantation and developmental biology.

## Methods

### Mouse stem cells for differentiation

ES cell lines used were YC5/EYFP cells derived from the totipotent cell line R1 [15,16] which carry the gene for enhanced yellow fluorescent protein (*EYFP*) under the control of a cytomegalovirus enhancer coupled to the *beta-actin *promoter (a gift from Andras Nagy); *Sox1-GFP *ES cells (a gift from Austin Smith and from Marios Stavridis) that contain a *GFP *reporter gene under the control of a *Sox1 *promoter [14]; and *tau-GFP *ES cells (a gift from John Mason) that carry the gene for *a tau-GFP *fusion protein [17]. The stem cells were propagated on gelatin-coated plates in ES cell medium consisting of DMEM (Gibco) containing 15% FBS (Gibco), 100 mM MEM nonessential amino acids (Gibco), 0.55 mM 2-mercaptoethanol, L-glutamine, and leukemia inhibitory factor (1000 units/ml; Chemicon). Early passage cells were frozen for later use.

### Formation of neural progenitor cells

Murine ES cells were thawed and grown to confluence prior to trypsinization for 3 min until detached cells were visible. The trypsin was inactivated by addition of 10% FBS in DMEM/F12 (1:1) and the cells were centrifuged for 5 min at 850 rpm and plated on non-tissue culture coated dishes in DMEM containing N2 supplement (Gibco). The cells were cultured on Petri dishes in DMEM supplemented with N2, and penicillin-streptomycin. Floating spheres formed in suspension and were examined for reporter-based fluorescence (*Sox1*) during culture for the next 7 d.

### Expansion of neural progenitor cells and neuronal differentiation

The spheres that were non-adherent at the end of the culture period were collected by centrifugation at 850 rpm for 5 min. The culture medium was aspirated and the cells were trypsinized for 3 min at 37°C with shaking. Trypsin was inactivated with 10% FBS in DMEM/F12 and the cells were collected by centrifugation at 850 rpm for 5 min. After removal of the supernatant by aspiration the cells were suspended in DMEM/F12 containing 10% FBS and plated on gelatin-coated and tissue culture-coated plastic dishes. After overnight attachment, the medium was removed and replaced with DMEM/F12 supplemented with N2 and B27 (Gibco) in the recommended concentrations and containing bFGF (20 ng/ml) and penicillin/streptomycin. They were grown to confluence and passaged every 3 d.

These neural progenitors were differentiated by growth in DMEM/F12 (1:1) containing N2 and B27 after removal of bFGF. The cells were cultured in this medium with replacement of half the medium every 3 d for time periods given in the text and monitored for loss of *Sox1-GFP *based fluorescence and gain of *tau-GFP *based fluorescence.

### Culture of cells for electrophysiological recordings

Frozen progenitor cells were thawed at 37°C and centrifuged at 850 rpm for 5 min. The supernatant was discarded and the cells were washed once in DMEM containing 10% FBS and plated as above. After overnight culture, the medium was replaced with fresh DMEM/F12 with bFGF and the medium was refreshed every other day.

The cells were trypsinized after reaching confluence and plated onto coverslips pretreated with 0.1% fibronectin. Differentiation was initiated by culture overnight in DMEM/F12 containing 10% FBS followed by culture in the medium above without bFGF. Half the differentiation medium was refreshed every 3 d, and the cells were allowed to differentiate for time periods given in the text.

### Semiquantitative RT-PCR

Total RNA was extracted with an RNeasy Minikit (Qiagen, Valencia, CA) according to the manufacturer's instructions. For reverse transcription, 6 μg of total RNA was used with SuperScript III transcriptase (Invitrogen) and oligo-dT primers. The PCR cycling conditions were optimized in pilot experiments. Specific cycling parameters were: initial denaturation step at 94°C for 2 min, followed by cycles of denaturation at 94°C for 30 s, annealing temperature optimized between 56-60°C for 30 s, extension at 72°C for 60 s, followed by 7 min of terminal extension at 72°C after the last cycle. The number of cycles was optimized between 30 and 35, and conditions were kept constant for each primer. The presented data are from experiments repeated at least 3 times. Control PCR without reverse transcriptase did not produce specific bands. The primer pairs and cDNA product lengths were:

*Oct4*, forward: ATG GCT GGA CAC CTG GCT TCA G; reverse: TTA ACC CCA AAG CTC CAG GTT C; 1033 bp; *Otx2*, forward: CCA TGA CCT ATA CTC AGG CTT CAG G; reverse: GAA GCT CCA TAT CCC TGG GTG GAA AG; 211 bp; *Sox2*, forward: CAC CCG GGC CTC AAC GCT CAC G; reverse: TCC CCT TCT CCA GTT CGC AGT CCA; 414 bp; *Pax2*, forward: CCA AAG TGG TGG ACA AGA TTG CC; reverse: GGA TAG GAA GGA CGC TCA AAG AC; 544 bp; *Pax6*, forward: AGA CTT TAA CCA AGG GCG GT; reverse: TAG CCA GGT TGC GAA GAA CT; 589 bp; *nestin*, forward: AAC AGA GAT TGG AAG GCC GCT GGC; reverse: CTT CAG AAA GGC TGT CAC AGG AG; 392 bp; *Musashi*, forward: ATG GAG ACT GAC GCG CCC CAG; reverse: ATC TTC TTC GTC CGA GTG AC; 332 bp; *Ngn1*, forward: TGG TGT CGT CGG GGA AC; reverse: AAG GCC GAC CTC CAA ACC TC; 400 bp; *Math1*, forward: AGA TCT ACA TCA ACG CTC TGT C; reverse: ACT GGC CTC ATC AGA GTC ACT G; 449 bp; *TrkC*, forward: ACC CGC ATC CCA GTC AT; reverse: TCC CGG TGT ACA AAG TGC; 521 bp; *jagged2*, forward: GTC CTT CCC ACA TGG GAG TT; reverse: GTT TCC ACC TTG ACC TCG GT; *GAPDH*, forward: AAC GGG AAG CCC ATC ACC; reverse: CAG CCT TGG CAG CAC CAG; 442 bp.

### Electrophysiology

Differentiated cells were placed into a recording chamber and viewed with a Zeiss Axioskop FS equipped with a 63× water-immersion lens and DIC optics. Recording pipettes were pulled from borosilicate capillary glass (R-6, Garner Glass) with resistances that ranged from 3-7 MW. The pipette tips were coated with ski wax to reduce pipette capacitance.

For electrophysiological recordings, cells were bathed in a standard extracellular solution that contained (in mM): 140 NaCl, 5 KCl, 1.3 CaCl_2_, 1 MgCl_2_, 10 HEPES, vitamins (1:100) and amino acids (1:50; Invitrogen, Carlsbad, CA) as in MEM. The solution was adjusted to pH 7.4 with NaOH and an osmolarity of 303 mOsmol/kg. Recording pipettes were filled with an intracellular solution containing (in mM): 135 KCl, 2.5 Mg-ATP, 0.1 CaCl_2_, 3.5 MgCl_2_, 5 ethylene glycol-bis (b-aminoethyl ether) -*N,N,N',N'*-tetraacetic acid, and 10 HEPES, adjusted to pH 7.4 with KOH and an osmolarity of 291 mOsmol/kg.

The whole-cell, tight-seal recording technique was used in both voltage- and current-clamp modes. All cells were held at -84 mV and data were acquired at room temperature (22-24°C) using an Axopatch 200B amplifier (Axon Instruments, Foster City, CA), filtered at 1 kHz with a low pass Bessel filter, digitized at 5 kHz with a 12-bit acquisition board, and acquired using pClamp 8.0 software (Axon Instruments).

All membrane potentials were adjusted for a 4 mV junction potential. Analysis was then performed using both the Clampfit 8.1 software (Axon Instruments) and Origin 7.1 (MicroCal Software, Northampton, MA). Data are presented as the mean ± standard deviation, unless otherwise noted.

To determine the range of activation and inactivation for K^+ ^and Na^+ ^conductances, we used a Boltzmann equation of the form:



*G*_*max *_and *G*_*min *_represented the maximum and minimum conductances respectively, *V*_1/2 _was the half maximal voltage, and *S *represented the slope factor.

### Immunohistochemistry

Floating spheres were fixed with 4% paraformaldehyde in PBS for 10 min. After 3 washes with PBS cells were permeabilized and nonspecific binding sites were blocked with PBT1 solution (0.1% Triton X-100, 1% BSA (w/v) and 5% heat-inactivated goat serum in PBS) for 20 min. The spheres were frozen in OCT and cryosectioned. Fixed and permeabilized sections were incubated with primary antibodies overnight in antiserum diluted in PBT1. Dilutions used were 1:500 for mouse monoclonal TuJ antibody (β-III tubulin, Covance), 1:200 for polyclonal rabbit anti-neurofilament M (145 kD) (Chemicon International), 1:1,000 for mouse monoclonal antibody to NeuN (Chemicon International), 1:500 for rabbit polyclonal antibody to GFAP (Dako), 1:1,000 for rabbit polyclonal antibody to NeuroD (Chemicon International), 1:1,000 for goat polyclonal antibody to Sox1 (R&D Systems), 1:1,000 for mouse monoclonal antibody to nestin (Developmental Studies Hybridoma Bank), 1:500 for rabbit polyclonal antibody to peripherin (Chemicon International), 1:50 for mouse monoclonal antibody to O4 (R&D Systems), and 1:1,000 for mouse monoclonal antibody to tyrosine hydroxylase (Sigma). Specimens were washed 3 times for 15 min each with PBS. Anti-rabbit and anti-mouse secondary antibodies conjugated to FITC, TRITC and Cy5 (Jackson ImmunoResearch) were used to detect primary antibodies. Cell nuclei were stained by exposure to 4,6-diamidino-2-phenylindole. Labeled cells were counted as a percentage of DAPI-stained nuclei (reported as mean ± SD). Staining was visualized with epifluorescence microscopy (Axioskop 2 Mot Axiocam, Zeiss) or confocal microscopy (TCD, Leica).

## Results

### Formation of neural progenitors in a defined medium

**Figure 1:**
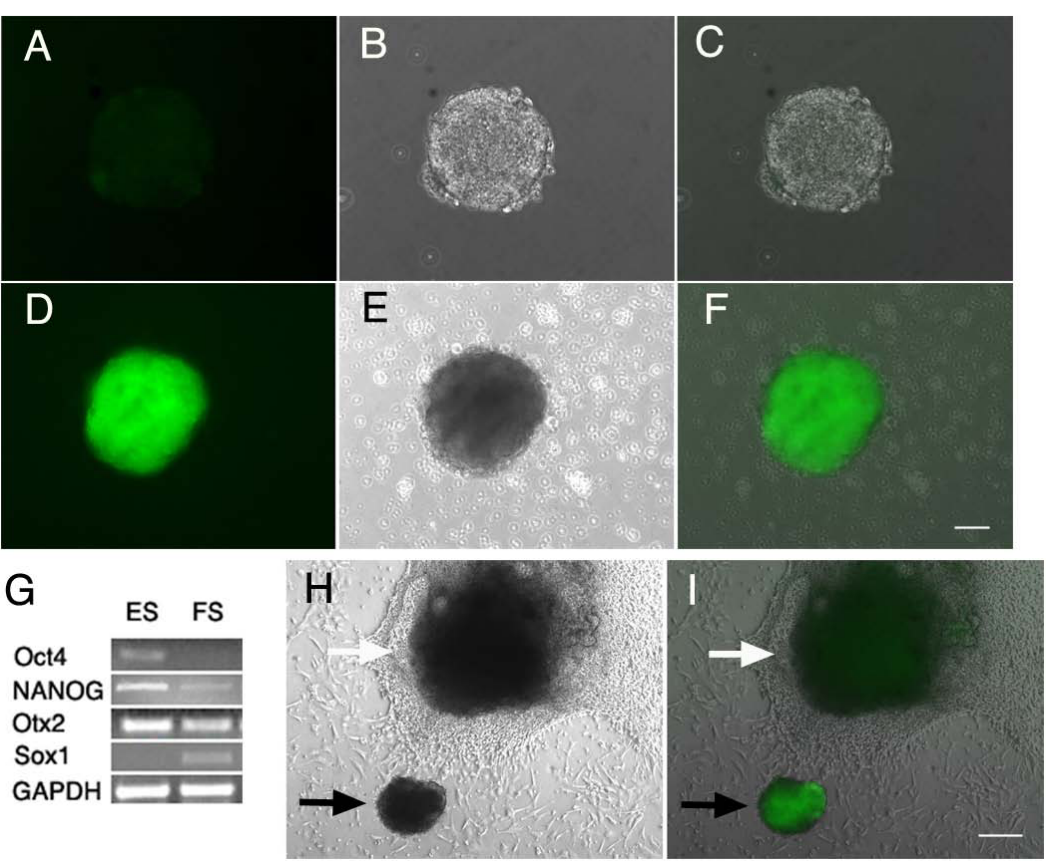
**Culture of embryonic stem cells in serum-free conditions**. The *Sox1-GFP *cells gave rise to bright green fluorescence in spheres that remained unattached during culture on non-tissue culture-coated plastic. The floating spheres on a Petri dish at day 1 of culture did not fluoresce (A is the GFP channel; B is phase; and C is the merged image) but became uniformly bright green 4 d after the start of culture (D is the GFP channel; E is phase; and F is the merged image). Expression of stem cell markers was assessed by RT-PCR (G). Stem cell markers *Oct4 *and *NANOG *decreased after differentiation of ES cells to neural progenitors in floating spheres (FS). *Otx2 *a marker for early placodal and forebrain neurons was maintained. *Sox1 *expression was first apparent at the time that neural progenitors formed. Floating spheres (I, black arrow) were fluorescent while attached spheres (I, white arrow) were not (H is the phase image and I is the merged phase and GFP images). Scale bars are 50 microns.

Mouse ES cells containing the GFP gene under the control of the *Sox1 *promoter [14] were trypsinized and dispersed into serum-free medium with N2 and B27 additives on an uncoated dish. The ES cells lacked green fluorescence, and floating spheres that formed in these culture conditions were not fluorescent after a 1 d floating culture (Figure 1A-C) but exhibited bright green fluorescence after 4 d in culture (Figure 1D-F). Most of the cells that remained in small clumps attached to the dish did not become fluorescent (Figure 1H, I; white arrow), but the cells in the aggregates referred to as floating spheres showed high expression of the *Sox1 *reporter (Figure 1H, I; black arrow). Comparison of the starting ES cells to the cells in the floating spheres after a 4 d culture revealed that the cells in spheres lost expression of stem cell marker *Oct4 *completely (Figure 1G), had decreased expression of *NANOG*, and continued to express the neuronal progenitor marker, *Otx2*. In agreement with the expression of the *Sox1 *reporter, mRNA for *Sox1 *was not found in ES cells, but was observed in the neural progenitors obtained at the end of this culture period (Figure 1G).

**Figure 2:**
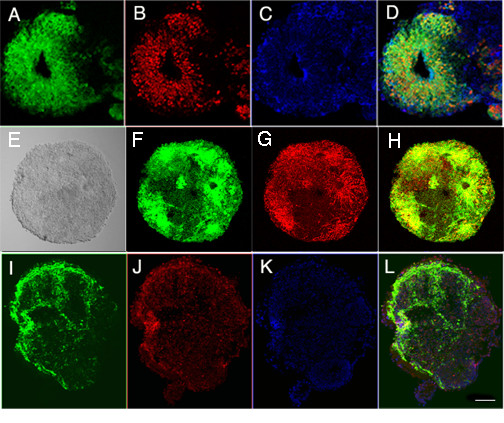
**Expression of neural progenitor markers in floating spheres**. Fluorescence from the *Sox1 *reporter (A, Sox1, green) correlated with nuclear Sox1 expression detected with an antibody (B, *Sox1*, red). DAPI staining is shown (C) and co-staining by the two methods is shown in the merged image (D). *Nestin *expression (G, red) also increased in the *Sox1 *positive spheres (F, *Sox1*, green). The overlap in labeling is shown in the merged image (H). *NeuroD *was expressed (I, green) in spheres along with peripherin (J, red). DAPI is shown in blue (K) and the overlap in labeling is shown in the merged image (L). Scale bar is 50 microns.

**Figure 3:**
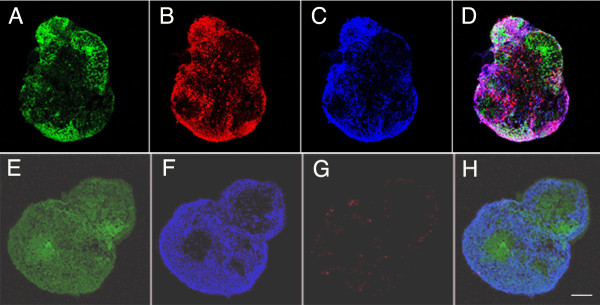
**Neural markers and glial markers were expressed in early spheres**. The *Sox1*-positive floating spheres (A, *Sox1-GFP*) were positive for βIII-tubulin (B, red) and neurofilament M (C, blue) indicating early upregulation of neural genes in the neural progenitor cells. The tubulin and neurofilament labeling overlap (D, merged). GFAP (G, in red) is expressed at the same time as β-III tubulin (F, in blue) in these *Sox1-GFP *containing spheres (E, in green), indicating that differentiation of both neural and glial lineages has been initiated in the neural progenitors. Most of these cells were non-overlapping (H, merged images). Scale bar is 50 microns.

The green fluorescence was shown to correspond to *Sox1-GFP *expression by staining of nuclear *Sox1 *in cells that had GFP expression in the cytoplasm (Figure 2A-D). Neuronal lineage markers including *nestin *(Figure 2E-H), *peripherin*, and *NeuroD *(Figure 2I-L) were expressed in the floating spheres. Cells in *Sox1*-positive spheres expressed markers of both neurons and glia, as shown by immunolabeling for tubulin and neurofilament (Figure 3A-D) and GFAP (Figure 3E-H).

**Figure 4:**
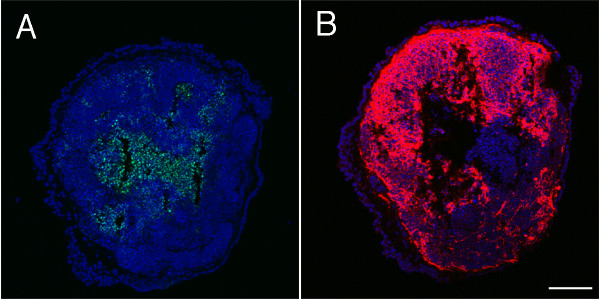
**Apoptotic cells were found in discrete clumps in areas of neurogenesis in the floating spheres**. Apoptotic cells (A, TUNEL in green) were expressed in cells adjacent to neural progenitor markers in the floating spheres (B, β-III tubulin in red) indicating that spheres that have been treated to induce formation of neural progenitors contain areas of apoptosis. DAPI is shown in blue. Scale bar is 50 microns.

Staining of the spheres for apoptotic markers showed that regions of the spheres underwent programmed cell death in serum-free medium. Apoptosis was detected in all floating spheres and the spheres gave rise to cells that expressed β-III tubulin (Figure 4). Staining for cell death in the spheres indicated that apoptotic cells (Figure 4A) were in close proximity to β-III tubulin-positive cells (Figure 4B). This observation suggested that the adjacent cells might have an influence on the generation of ectoderm and the formation of neuronal precursors. We hypothesize based on our recent data in human ES cells [18] that cell death may have been due to selective pressure of the serum-free medium that did not permit survival of non-ectodermal cells.

### Propagation of progenitors in bFGF and neural differentiation

**Figure 5:**
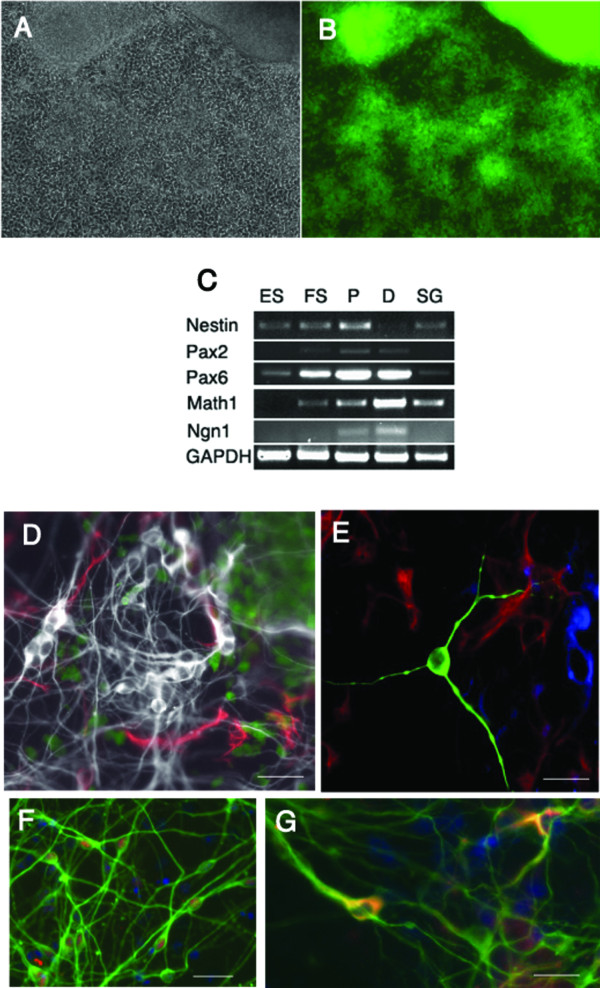
**Attachment and differentiation of neural progenitors**. Neural progenitors were expanded in the presence of FGF to obtain increased numbers of cells. Progenitors that remained attached to the tissue culture plate after 3 d maintained their expression of *Sox1 *(phase contrast in A; *Sox1-GFP *in B) as well as other neural progenitor markers, *nestin*, *Pax2 *and *Pax6 *(C) which were expressed in both the floating (FS) and proliferating progenitors (P) that were expanded in FGF as detected by RT-PCR. *Math1 *and *Ngn1 *were expressed starting at the neural progenitor stage and were present in differentiated cells (D) as well. Neurons from the spiral ganglion (SG) of a newborn mouse are shown for comparison. Low power image of neural lineage cell types formed after culture without FGF in the monolayer for 7 d is shown in D. Cells were positive for *Sox1-GFP *(shown in green), β-III tubulin (shown in white), and GFAP (shown in red). A high power image shown in E is positive for neural (β-III tubulin staining shown in green), glial (GFAP staining shown in red), and oligodendrocyte markers (O4 staining shown in blue). Neurons obtained after 10 d could be detected with NeuN (red) and β-III tubulin antibodies (green) in F. The neurons formed in the mixture included neurons with tyrosine hydroxylase expression (G, in red) as well as β-III tubulin (G, in green). DAPI is shown in blue.

The neural progenitors obtained in the floating spheres were expanded by growth in a monolayer. When the neural progenitors were cultured on tissue culture plastic, the spheres attached and grew in combination with cells that grew out as a monolayer from the islands (Figure 5A). We tested the expression of markers in the cells after they had attached to plastic and been cultured in medium containing bFGF for 3 d. The cells maintained their expression of neural progenitor markers such as *Sox1 *based on fluorescence (Figure 5B). Both attached cells and floating spheres from which they were derived showed expression of neuronal precursor markers such as *nestin *(Figure 5C). They also maintained expression of markers of neuronal ectoderm, *Pax2 *and *Pax6*. They increased expression of DRG and placodal progenitor markers, *Ngn1 *and *Math1*, respectively (Figure 5C). These markers reveal that the expanded progenitors had the capacity to develop into several neuronal lineages.

Induction of neural differentiation by the removal of growth factors gave rise to neurons, glial cells and oligodendrocytes (Figure 5D-G). Neurons with cytoplasmic staining for β-III tubulin were obtained in high yield (90.8 ± 1.0% of 8145 cells counted), and NeuN expression was detected in their nuclei (Figure 5F), while glial cells (Figure 5D) staining for GFAP and oligodendrocytes staining for O4 constituted less than 10% of the total cells (Figure 5E). These ratios varied with the length of time taken for differentiation. The populations of neurons included cells that were developing as dopaminergic types (4.8 ± 0.3% of 2275 cells) as revealed by tyrosine hydroxylase staining (Figure 5G).

### Fluorescent reporters in the differentiated neurons

**Figure 6:**
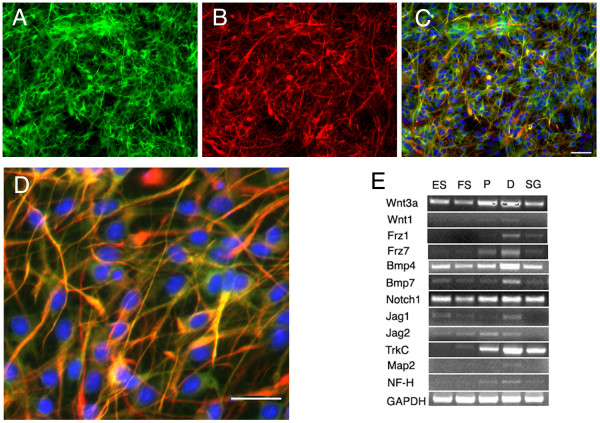
**A neuronal reporter is expressed in differentiated cells obtained from ES cells**. ES cells expressing GFP as a fusion protein with tau were fluorescent after differentiation for 10 d to cells with the morphology of neurons (A, in green) and stained for β-III tubulin (B, in red). Merged images are shown in low power in C and high power in D with scale bars indicating 50 microns. Both floating spheres (FS) and attached proliferating cells (P) are shown in E to express an array of developmental regulators (*Wnt3a*, *Wnt1*, *Frz1*, *Frz7*, *BMP4*, *BMP7*, *Notch1*, *Jag1*, *Jag2*). Neurons differentiated from neural progenitors (D) had similar expression of neuronal markers, *NF-M*, *TrkC *and *MAP2 *as spiral ganglion neurons (SG).

ES cells engineered to express a tau-GFP fusion protein were not fluorescent at the ES cell or neural progenitor stage, but after differentiation to neurons, the cells showed strong expression of tau-GFP (Figure 6A-D). Outgrowth of neurites from the cells was detected readily because of the intense labeling of neurites (Figure 6D). Cells expressing this reporter also displayed expression of *neurofilament-H*, *TrkC *and *MAP2 *in the cells differentiated from progenitors (Figure 6E). These progenitors and the differentiated neurons expressed a wide array of developmental regulators (*Wnt3a*, *Wnt1*, *Frz1*, *Frz7*, *BMP4*, *BMP7*, *Notch1*, *Jag1*, *Jag2*) similar to regulatory molecules expressed by newborn sensory ganglia.

### Functional characteristics of differentiated stem cells

**Figure 7:**
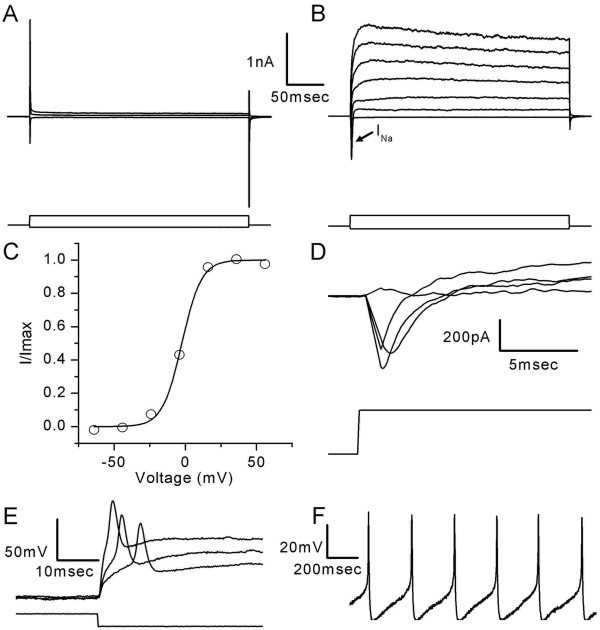
**Differentiated stem cells display neuronal characteristics**. Currents recorded from an undifferentiated cell (A) (upper panel) in response to voltage steps to -124, -14 and 66 mV (lower panel). A representative family of currents (B) (upper panel) recorded from a cell differentiated for 20 d. Both inward Na\+(+) and outward K\+(+) currents were observed. The lower panel shows the voltage protocol with steps to (mV): -124, -24, -4, 16, 36, 56, 76. An activation curve (C) that shows the voltage generated from a Boltzmann fit to data from the cell shown in panel B (V\-(1/2) = -2.4 mV; S = 6.8 mV). Fast activating, fast inactivating inward Na\+(+) currents (D) recorded from a differentiated stem cell (upper panel). The lower panel shows the voltage protocol with a prepulse step to -104 mV followed by a family of steps to (mV) -29, -19, -14, -4. A representative family of voltage responses recorded in current-clamp mode (E) from a differentiated cell (upper panel). The lower panel shows the current protocol with steps to (pA): 0, -200, -600. In one cell we noted spontaneous action potentials in the absence of stimulation (E). All cells were held at -84 mV.

In order to assess the functionality of cells that had been through the differentiation, whole-cell, tight-seal recordings were taken. Cells were allowed to differentiate *in vitro *for up to 20 d. We recorded both voltage-dependent currents in voltage-clamp mode and membrane potentials in current-clamp mode. We recorded from a total of 57 cells: 14 of these displayed no current (Figure 7A), 23 exhibited neuronal-like K^+ ^currents, while the remaining 20 cells had both K^+ ^and Na^+ ^currents with neuronal characteristics (Figure 7B and 7D). Furthermore, we found that in current-clamp mode, the differentiated cells generated action potentials (Figure 7E and 7F). Of the 43 cells that expressed voltage-dependent currents, we selected data from 10 cells for more quantitative analysis based on the quality of the recording (i.e., low pipette leak and low series resistance).

To analyze the properties of the voltage-dependent K^+ ^currents in the neuronal cells we examined them in voltage-clamp mode. We applied voltage steps that ranged from -124 to 76 mV every 10 mV, which evoked slowly activating outward currents that did not inactivate. The mean peak amplitude of the currents was 2.02 ± 0.97 nA at 76 mV (n = 5) (Figure 7B). The mean reversal potential of the outward K^+ ^current was calculated by applying a depolarizing step to 16 mV to activate the outward current, followed by test steps that ranged from -104 to -14 mV in 5 mV intervals. The tail currents were measured immediately after the test step and were plotted as a function of test potential to determine the mean reversal potential (-56 ± 16 mV; n = 5), which was similar to the potassium equilibrium potential. We also examined the voltage range of activation for the K^+ ^currents by applying voltage steps that ranged from -124 to 96 mV every 20 mV, followed by a step to -54 mV. The tail currents observed at the instant of the step to -54 mV were recorded and divided by driving force to generate conductance. Conductance was plotted as function of prepulse potential and fitted with a first order Boltzmann equation (Figure 7C). The mean V_1/2 _of activation for the K^+ ^currents was -6.8 ± 5.4 mV with a slope of 7.3 ± 4.4 mV (n = 5) and the mean maximal conductance was 2.02 ± 0.77 nS (n = 5).

The cells also expressed fast activating, fast inactivating Na^+ ^currents (Figure 7D). To analyze these currents, a 100-msec prepulse to -104 mV was applied to relieve Na^+ ^channel inactivation, followed by voltage steps that ranged from -84 to 11 mV every 5 mV. The protocol evoked small inward Na^+ ^currents with mean peak amplitudes of -396 ± 230 pA (n = 5). The mean V_1/2 _of activation was -22.9 ± 1.6 mV with a slope of 3.2 ± 0.3 mV (n = 5). Na^+ ^inactivation was also examined by applying voltage steps ranging from -129 to -29 mV in 5 mV intervals for 50 msec followed by a step to -44 mV. We recorded the peak current evoked by the step to -44 mV and plotted these data as a function of prepulse potential. We fit the data with a Boltzmann equation and observed a V_1/2_of inactivation of -54.7 ± 2.8 mV with a slope of 2.6 ± 1.7 mV (n = 3).

In order to study the membrane potential characteristics of the neuronal cells, we used current-clamp mode. Action potential generation was observed in 6 of the 20 cells that exhibited both K^+ ^and Na^+ ^currents. In all 6 cells, action potentials were evoked in response to 300-msec current injections that ranged from -200 to 600 pA (Figure 7E). In one case the cell fired spontaneously in the absence of stimulation (Figure 7F). The mean resting potential of the cells was -38.2 ± 11.7 mV (n = 10).

Another population of cells was examined which displayed only outward K^+ ^currents and had neither Na^+ ^currents nor generated action potentials. The voltage-dependent properties of these cells were analyzed using the same protocols as mentioned previously. The mean peak K^+ ^current amplitude measured at 76 mV was 1.5 ± 2 nA (n = 5). Using a first order Boltzmann equation to fit the K^+ ^conductance values, we calculated the V_1/2 _of activation to be -4.3 ± 13.3 mV with a slope of 6 ± 3.3 mV (n = 5). As these cells only displayed outward K^+ ^currents, we suspect that they were immature neurons and that had they been allowed longer culture times they would have expressed Na^+ ^currents and acquired the ability to generate action potentials.

## Discussion

We describe a new method for the conversion of ES cells to neuroectoderm progenitors that uses floating spheres to induce the ES cells to develop into neural progenitors and serum-starvation to inhibit the development of other embryonic progenitors in order to achieve production of the desired progenitor cells in high yields. This method has the advantage that the differentiation of the cells and loss of pluripotency is well-controlled, giving rise to a cell population that is primarily of neuroectoderm lineage. The formation of neural progenitors appears to be aided by programmed cell death of adjacent cells in the floating spheres.

Compared to methods that use formation of embryoid bodies prior to selection for neural lineage, the method is rapid and requires less intensive cell culture. Both neuronal lineage specification and generation of functional neurons occur in 17 d, with 7 d to form the floating spheres containing neural progenitors and 10 d to obtain neurons. The protocol does not require feeder cells and is less complicated than those requiring rosette formation and selection of individual colonies by hand prior to expansion of progenitors [1,5,8,9]. Neural lineage specification is presumably assisted by the formation of spheres, suggesting a similarity to the organization of neurospheres that are readily formed from adult neural stem cells [19]. Enrichment of cells that formed neurons upon subsequent differentiation was also observed in a method that used spheres but maintained the spheres in a pluripotent state by inclusion of leukemia inhibitory factor (LIF) in the medium [20]. Methods involving monolayer culture [14] for generation of neural progenitors are also rapid and simple but may not result in the same numbers of neurons as obtained in the floating spheres, and a method using floating culture for human ES cells was less efficient for the generation of neurons than the method described here [21]. The monolayer culture method is not applicable to human ES cells [22] since the cells formed clumps and the neural cells arose in these clumps. Compared to methods that use low density culture in the presence of LIF [23] to generate floating spheres, the method described here is independent of added growth factors. This is presumably due to the higher cell density in the spheres that we use for generation of the neural progenitors, which takes advantage of endogenous paracrine factors, as shown in a recent study that also used spheres. In that study, spheres generated in the presence of N2 supplement and LIF contained intermediate progenitors that were competent for neurogenesis but expressed ES cell markers [20]. This was unlike the spheres in our study, which lose expression of ES cell markers upon their conversion to progenitors in the serum-free medium which contains N2 supplement but does not contain LIF. The low-density methods are not applicable to human ES cells that require higher cell density for survival. We have recently shown in a separate paper [18] that our method is applicable to human ES cell lines using the same procedures. The reporter cell lines that we used detected the initial conversion of these cells to neural progenitors as well as the subsequent differentiation of these cells to neurons.

The formation of the neural progenitors followed pathways that were identical to those seen *in vivo *and these cells can therefore be used to study neuronal differentiation. They gave rise to neural lineage cells expected from neural progenitors including neurons, glia and oligodendrocytes. During the *in vitro *differentiation of the ES cells to these progenitors, *Oct4 *was observed to decrease as *Pax2 *and *Pax6 *increased. The expression of *Pax6 *preceded that of *Sox1*, as observed for precursors of motor neurons [9]. As in the developing otic placode, the expression of *Math1 *was upregulated after that of both *Otx2 *and *Pax2*. We obtained neurons that could be detected by expression of *Pax2*, *NeuroD*, *TrkC*, and *Brn3a *all of which are expressed in developing neuroepithelium. At the same time that these neuronal specification genes were upregulated, the developmental regulators, *BMP4 *and *BMP7 *continued to be expressed as has been observed by others in stem cell derived progenitors [1,8-10,22,24], and *Wnt *pathway genes were increased. Expression of these markers showed that the neural progenitors had the capacity to develop into several neuronal lineages and this was illustrated further by the differentiation of dopaminergic neurons identified by tyrosine hydroxylase staining [1,6].

The apoptosis of progenitors is apparently a required step in the high yield of neuroectoderm achieved. Reduction in endoderm and mesoderm markers in the course of the maturation of the spheres was apparent in our recent work [18] and the accompanying increase in *nestin *and *Pax2 *and *Pax6*, markers of neural progenitor cells, is indicative of the formation of neuroectoderm lineage in the spheres as previously observed [23]. The close apposition of dying cells with the newly formed neural lineage is similar to the programmed cell death that accompanies the development of the embryonic nervous system [25], and the differentiation of the neural progenitor cells may be driven by inductive signals emanating from dying cells. The cells that undergo apoptosis are most likely from nonneuronal lineages that don't survive under the culture conditions used.

The neurons formed from these neural progenitors could be induced to generate action potentials and were, therefore, functional neurons that could potentially be used for replacement of cells lost to neurodegeneration. The neuronal phenotype that we have observed *in vitro *has prompted us to use the neural progenitors for studies of neuronal replacement in an *in vivo *model, and we found [18,26] that neurons differentiated from mouse and human ES cells integrated into the host tissue. The neurons will be valuable in cell transplantation applications based on preliminary results (data not shown) that the neural progenitors make synaptic vesicles at their growing tips and express axonal markers upon contact with sensory hair cells of the inner ear in a cultured explant of the cochlea [27].

## Conclusion

Neurons and glial cells were differentiated from ES cells by a method that initially produced neuroectoderm by a combination of nonadherent conditions and serum starvation. Neurons are obtained in high yield in 17 d, with 7 d to form the floating spheres and 10 d for neural progenitors to differentiate into neurons. The neurons obtained stain for neuronal markers and are functional. The protocol does not require feeder cells and is less complicated than those requiring rosette formation and selection of individual colonies. Neural lineage specification is presumably assisted by the formation of spheres, similar to those formed from neural stem cells.

## Abbreviations

EYFP: enhanced yellow fluorescent protein; GFP: green fluorescent protein; ES: embryonic stem.

## Authors' contributions

Original concept was by HL, SH, and ASBE. Differentiation experiments were performed by HL, HL, CEC, and JF. PCR experiments were performed by JF. Electrophysiology was performed by JRR and JRH. Manuscript was written and edited by HL, CEC, JRR, JF, JRH, SH, and ASBE. All authors read and approved the final manuscript.
